# Confined Polymers as Self-Avoiding Random Walks on Restricted Lattices

**DOI:** 10.3390/polym10121394

**Published:** 2018-12-15

**Authors:** Javier Benito, Nikos Ch. Karayiannis, Manuel Laso

**Affiliations:** Institute for Optoelectronic Systems and Microtechnology (ISOM) and ETSI Industriales, Universidad Politécnica de Madrid, José Gutiérrez Abascal, 2, 28006 Madrid, Spain; javier.benito.piedra@alumnos.upm.es (J.B.); nkarayiannis@etsii.upm.es (N.C.K.)

**Keywords:** freely jointed chain, confinement, enumeration, conformational entropy, phase transition, self-avoiding random walk, face-centered cubic, simple cubic, lattice model, hard sphere

## Abstract

Polymers in highly confined geometries can display complex morphologies including ordered phases. A basic component of a theoretical analysis of their phase behavior in confined geometries is the knowledge of the number of possible single-chain conformations compatible with the geometrical restrictions and the established crystalline morphology. While the statistical properties of unrestricted self-avoiding random walks (SAWs) both on and off-lattice are very well known, the same is not true for SAWs in confined geometries. The purpose of this contribution is (a) to enumerate the number of SAWs on the simple cubic (SC) and face-centered cubic (FCC) lattices under confinement for moderate SAW lengths, and (b) to obtain an approximate expression for their behavior as a function of chain length, type of lattice, and degree of confinement. This information is an essential requirement for the understanding and prediction of entropy-driven phase transitions of model polymer chains under confinement. In addition, a simple geometric argument is presented that explains, to first order, the dependence of the number of restricted SAWs on the type of SAW origin.

## 1. Introduction

Self-avoiding random walks (SAWs) have long been used in polymer science as one of the simplest and most useful descriptions of polymeric chains. The relative simplicity of SAWs has made them an ideal tool to investigate static and dynamic properties of polymers both analytically and computationally [1,2,3,4,5,6,7] . They have proved particularly useful in the determination of universal behavior and scaling laws for polymer systems ranging from individual chains to melts. The critical behavior of SAWs is also closely related to that of the Ising model and to percolation [8,9,10,11,12,13,14,15,16,17,18].

Besides their extensive application in polymer science, SAWs have been a subject of mathematical interest in their own right [19,20], mainly because of their close relationship to Brownian motion and stochastic processes in general soft matter physics [21,22,23]. In spite of the very simple idea underlying SAWs, comparatively few results have been rigorously solved in a mathematical sense [19]. As a consequence, a great deal of computational work has been carried out to complement analytical approaches. From the numerical point of view, a currently active research area is the efficient computation of the number of distinct conformations for an SAW of a given length on a lattice, which is very closely related to the single-chain classical partition function [24]. Over the last several years, increasingly sophisticated enumeration algorithms [25,26,27] have been continually pushing the upper SAW length limit for which numerical results on enumeration can be obtained within a reasonable computational time.

Detailed knowledge of SAW properties in restricted geometries is an essential ingredient in the study of confined polymeric systems, which can range from single macromolecules to highly entangled melts in pores, slits, narrow gaps and nanocavities. Such properties include the number of distinct SAWs for a given length, mean squared end-to-end vector, distribution of size, etc. Although SAWs in such restricted geometries have also been studied [11,12,28,29,30,31,32], they have received far less attention than unrestricted SAWs, one of the reasons being the apparent lack of applications in polymer science. The relatively recent [33,34,35,36,37,38,39,40,41,42,43,44,45,46,47,48] increased interest in confined polymeric systems, accompanied by significant advances in molecular simulations and the availability of experimental techniques able to probe the behavior of individual macromolecules in channels, slits, etc. [49,50,51,52,53] is a strong motivation for the investigation of SAWs in such confined geometries. Recent Monte Carlo (MC) simulations [54] of highly confined, dense assemblies of linear, freely jointed chains of strictly tangent hard spheres of uniform size show that such athermal polymer systems display an unexpectedly broad range of morphologies, presumably connected by phase transitions.

In the following, “polymer” will refer to a linear chain of strictly tangent hard spheres, unless explicitly stated otherwise. “Monomer” will refer to each of the hard spheres that make up a chain, and “site” will refer to each of the points of a lattice. We will also refer interchangeably to the cubic P and F lattices and the corresponding simple cubic (SC) and face centered cubic (FCC) crystals obtained by placing a spherical base motif on all lattice points.

**Figure 1:**
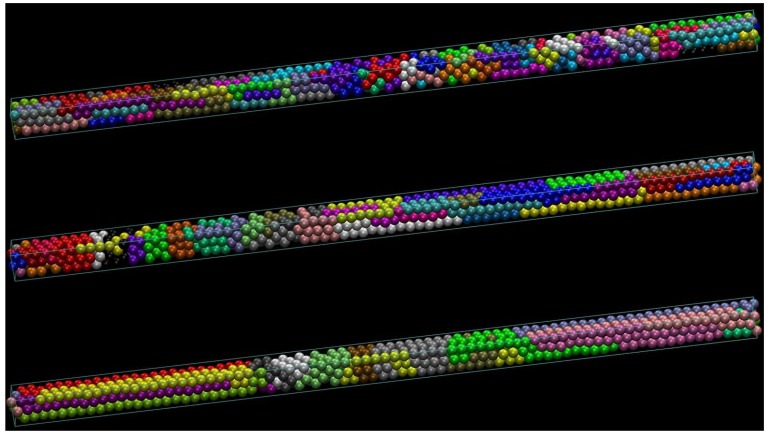
Lateral views of computer-generated, linear freely jointed chains of tangent hard spheres of uniform size confined in tubes of square cross section at φ=0.50. All systems contain a total of 720 monomers. From top to bottom: chains consist, on average, of N= 7, 17 and 35 bonds. In all cases, chains are packed in an approximately 3.11 × 3.11 square tube of length 77.8. Periodic boundary conditions are applied on the long dimension and impenetrable flat walls in the short ones. Ordered regions with crystalline defects can easily be recognized by visual inspection. A precise analysis shows them to be slightly defective, coexisting face-centered cubic (FCC) crystals of different orientations. Monomers have been colored according to the chain they belong to. The tube axis direction in both panels is along a direction of the crystallographic type 〈100〉. Image created with the VMD software (version 1.9.3, Theoretical and Computational Biophysics Group, University of Illinois, Urbana, IL, USA) [68].

**Figure 2:**
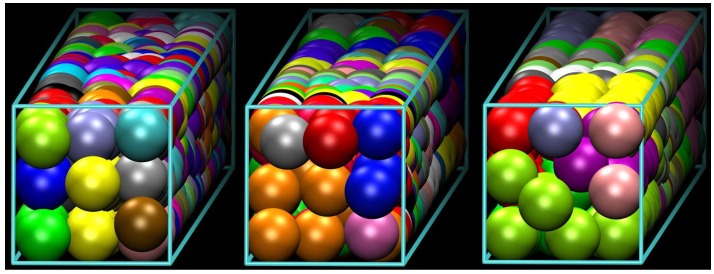
Same as in Figure 1 but for cross-sectional views. From left to right: chains consist, on average, of *N* = 7, 17 and 35 bonds.

As stated earlier, the present work is motivated by the simulation results of Ref. [54] where linear, freely-jointed chains of tangent hard spheres of uniform size are generated and successively equilibrated under various conditions of confinement. The latter is realized through the presence of flat, impenetrable parallel walls in one or more dimensions. Extreme confinement corresponds to the state where inter-wall distance approaches monomer diameter leading eventually to the formation of quasi 1D (tube-like) and 2D (plate-like) polymer templates. Typical computer-generated polymer configurations can be seen in Figure 1 and Figure 2 in lateral and cross-sectional views, respectively. They correspond to systems containing a total of 720 monomers and average number of bonds per chain N=7, 17 and 35 at a packing density φ=0.50. In all cases, chains are packed in an approximately 3.11 × 3.11 square tube of length 77.8. All lengths are reported in units of monomer diameter (equal to the SAW step length). Periodic boundary conditions are applied on the long dimension, and hard walls exist in the short ones. More details on the simulation algorithm, the systems studied and the corresponding model parameters can be found in [54].

Visual inspection of athermal chain configurations confined in square tubes, as the ones shown in Figure 1 and Figure 2, reveals the presence of highly ordered regions with crystalline defects. A more precise analysis of the local environment around each sphere monomer identifies such structures as slightly defective, coexisting FCC crystals of different orientations. Entropy is the sole driving force for structural transitions between different ordered morphologies in such athermal systems. Accordingly, an enumeration of all possible chain configurations on a specific regular lattice, subject to spatial restrictions arising from confinement, would allow us to determine the conformational component of entropy and eventually predict the stability of each distinct polymer crystal.

An analysis, based on the Characteristic Crystallographic Element (CCE) norm [55,56,57], of the geometrical environment around the spherical monomers shows the ordered regions in such highly-confined polymer structures to very closely correspond to an FCC crystal. One remarkable aspect of such dense polymer systems in the bulk (i.e., without spatial confinement) is the existence of highly ordered, crystalline phases [58]. In previous MC work [57,58,59,60,61,62,63,64], it was shown that the apparent loss of entropy, caused by the regular organization of monomers in the sites of a crystal lattice, is more than compensated for by the increase of available volume for monomers, and hence translational entropy, as made evident by sharp decreases in asphericity and acylindricity of the Voronoi cells associated with each monomeric site. The resulting crystalline structures strongly resemble those appearing in Molecular Dynamics (MD) and MC simulations of *single* (monomeric) spheres, well known since the pioneering work of Alder and Wainwright [65,66,67]. These crystalline *polymer* structures can be simplistically viewed as built from crystals of single hard spheres and overlaying on them all possible linear paths of a given length that connect tangent spheres. Vice versa, configurations of single hard spheres can be obtained trivially from available configurations of polymers by deleting all bonds in chains.

As a matter of fact, if chain connectivity is ignored and the monomers are considered as individual spheres, the resulting ordered structures are virtually indistinguishable, except for one main feature, from those appearing in single hard sphere systems [69,70,71]. The distinguishing feature is the absence of twinned structures in polymer systems [72]. In computer simulations, packings of single hard spheres often form quite perfect tetrahedral clusters which tend to aggregate in pentatwins [73]. The entropic conformational entropy loss associated with twinning in polymeric systems raises the entropic barrier to the extent that individual crystals with single or multiple stacking directions and abundant defects are observed predominantly in simulations.

Since difference in entropy is the only hindering or driving force for phase transitions in athermal polymeric systems [6,74,75,76], the entropy calculation in confined geometries is an essential requirement in understanding and predicting their phase behavior. Although all previously described characteristics have been obtained from off-lattice simulations, the appearance of highly ordered crystalline phases in quasi 1D (tube-like) confined polymer systems, such as the ones shown in Figure 1 and Figure 2, motivates the calculation of their entropy on crystal lattices under equivalent spatial restrictions.

**Figure 3:**
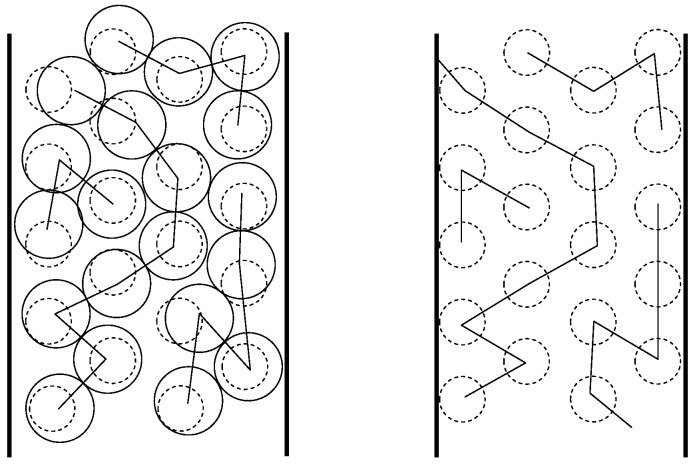
Schematic representation of ordered polymer structures in a confined geometry. Circles inside a solid line represent spherical monomers, polygonal lines represent polymer backbones. Monomers along a chain are strictly tangent (circles in solid line on left panel), monomers belonging to different chains do not need to, but can also be tangent. On both panels, circles with dashed lines represent sites of the perfect crystal. On average, polymer backbones can be considered self-avoiding random walks (SAWs) on the sites of the perfect crystal (right panel).

Figure 3 is a simplified, generic, two-dimensional representation of the ordered structures observed in MC simulations of highly confined polymeric systems [54]. The left panel represents a typical system configuration (MC-snapshot) confined between parallel walls. The centers of the spherical monomers (circles in solid line) are, on average, close to the sites of the perfect crystal (circles in dashed line). Configuration space is sampled through changes in the positions of the monomers as the MC progresses (such changes being compatible with chain connectivity, packing density, confinement and crystalline morphology; see, for example, the corresponding MC algorithms in [54,77]), much as monomer vibrations about the equilibrium position sample configurations in MD simulations. At high densities, monomers remain close to the sites of the crystal lattice (shown in the right panel), so that on-lattice polymer chains, built by joining the corresponding sites of the perfect crystal, closely approximate the original off-lattice system from the conformational point of view. Each of these chains is thus effectively a restricted SAW on the crystal lattice.

In typical classical MC simulations [78,79,80,81,82], configurations for off-lattice polymer systems are generated with a probability proportional to their statistical (Boltzmann) weight and correspond to individual points in a configuration space spanned by continuously varying degrees of freedom, e.g., Cartesian coordinates of monomer centers in an MD formulation based on Newton’s equations of motion, or Euler, torsion and bond angles in a Lagrangian formulation, etc. Entropy or free energy calculations require then the evaluation of a high-dimensional integral in configuration space [78].

On the other hand, configuration space for lattice SAWs (Figure 3, right panel) is discrete and entropy is evaluated as a sum of Boltzmann probabilities or weights. Since all feasible configurations are equally probable in athermal systems, entropy is proportional to the logarithm of the number of different SAWs. While extensive work on the exact enumeration of SAWs on unrestricted lattices in several dimensions (typically the *d*-dimensional hypercubic lattice $Z^{d}$) has been carried out, enumeration of SAWs on restricted cubic P and F lattices has not been reported to date. In this contribution, we evaluate, by direct enumeration, the number of SAWs on the cubic P and F lattices subject to geometrical restriction and calculate the SAW size as a function of lattice type, number of bonds and level of confinement.

## 2. Methods

In the following, an *N*-step three-dimensional SAW $\omega^{N}$ on a lattice is defined as the ordered sequence of sites ${\underline{\omega }}^{N}\left(0\right),{\underline{\omega }}^{N}\left(1\right),\ldots ,{\underline{\omega }}^{N}\left(N\right)$, where ${\underline{\omega }}^{N}\left(0\right)$ is the position vector of the SAW origin, satisfying the condition ${\underline{\omega }}^{N}\left(i\right)\neq {\underline{\omega }}^{N}\left(j\right)$ for i≠j, and such that $\left|{\underline{\omega }}^{N}\left(i+1\right)-{\underline{\omega }}^{N}\left(i\right)\right|=1,\;i\in \{0,1,\ldots ,N-1\}$, where it is assumed that the step length of the SAW is taken as the unit of length, and $|\underline{x}|=\sqrt{\underline{x}\cdot \underline{x}}$ denotes the usual Euclidean norm.

According to the previous definition of step length, two neighboring sites are 1 length unit apart on both the cubic P and the F lattices. For the cubic P lattice, the edge length of the conventional cell is therefore also a unit, whereas, in the cubic F lattice, the edge length of the conventional cell is $\sqrt{2}$.

The individual components of the position vector of the *i*-th site of an *N*-step SAW are denoted by $\omega_{j}^{N}\left(i\right)$ with j=1,2,3. The squared end-to-end distance of the SAW ${\left|\omega^{N}\right|}^{2}$ is given by ${\left|\omega^{N}\right|}^{2}=\left({\underline{\omega }}^{N}\left(N\right)-{\underline{\omega }}^{N}\left(0\right)\right)\cdot \left({\underline{\omega }}^{N}\left(N\right)-{\underline{\omega }}^{N}\left(0\right)\right)$. With the previous definitions of unit length, ${\left|\omega^{N}\right|}^{2}=N^{2}$ for a fully extended SAW, whereas the minimum SAW length is $\min({\left|\omega^{N}\right|}^{2})=1$. These two values bracket the range over which the distribution of ${\left(\omega^{N}\right)}^{2}$ is defined. If we denote by $c_{N}$ the number of distinct *N*-step SAWs, the average squared end-to-end distance is given by:

$$\begin{array}{c} \langle {\left|\omega^{N}\right|}^{2}\rangle =\frac{1}{c_{N}}\sum_{\omega^{N}}{\left|\omega^{N}\right|}^{2}, \end{array}$$

where the sum is over the $c_{N}$ SAWs starting at a given lattice point ${\underline{\omega }}^{N}\left(0\right)$. For unrestricted SAWs, ${\underline{\omega }}^{N}\left(0\right)$ can be any one of the countable infinity of lattice points, since the set $\left\{\omega^{N}\right\}$ of all SAWs starting at all points of a given lattice has the same space group symmetry as the lattice itself. Let us define the following equivalence relation on the set $\left\{\omega^{N}\right\}$ of all three-dimensional SAWs of a given length *N* starting at all points of a given lattice: two SAWs $\omega^{N}$, $\omega^{\prime }{}^{N}\in \left\{\omega^{N}\right\}$ are equivalent, and we write $\omega^{N}\approx \omega^{\prime }{}^{N}$, if there exists a geometrical transformation *T* (group element) in the space group $Ia\bar{3}d$ such that $T\left({\underline{\omega }}^{N}\left(i\right)\right)\approx {\underline{\omega }}^{\prime }{\left(i\right)}^{N}\;i\in \left\{0,1,\ldots ,N\right\}$. The set of all distinct $c_{N}$ SAWs is then the set of all equivalent classes $\left\{\omega^{N}\right\}/c_{N}$. For confined SAWs, the introduction of geometric restrictions will reduce this trivial multiplicity (which is due to the maximal symmetry of the unconfined lattice).

For unrestricted lattices, the number $c_{N}$ and thus the computational effort for the exact enumeration problem for SAWs are believed to grow exponentially with power law corrections as *N* increases, instead of the purely exponential growth for simple non-SAWs. More specifically, it is conjectured, and there is strong numerical and nonrigorous evidence, that $c_{N}$ and $\langle {\left|\omega^{N}\right|}^{2}\rangle$ depend on *N* as:

(1) $$\begin{array}{c} c_{N}∼A\mu^{N}N^{\gamma -1}, \end{array}$$

(2) $$\begin{array}{c} \langle {\left|\omega^{N}\right|}^{2}\rangle ∼DN^{2\nu }, \end{array}$$

where *A*, *D*, μ, γ and ν are (dimension dependent) positive constants. The constant *A* is known as the amplitude, μ as the connective constant, while γ (the entropic exponent) and ν are critical exponents. For simple non-SAWs, γ=1 and $\nu =\frac{1}{2}$. Estimates and bounds for μ, ν and γ for SAWs are available [25,83,84,85,86,87,88,89]. Approximate values in three dimensions are μ≈4.684, γ≈1.157 and ν=0.588.

The value of $c_{N}$ has been the object of increasingly refined and extensive calculations. Milestone calculations for the 3D cubic P lattice are: Orr’s N≤6 [24], Fisher and Sykes N≤9 [17,90,91], Guttmann N≤21 [83,88,91,92,93,94], MacDonald et al. N≤26 [84,88], Clisby et al. N≤30 [87], Schram et al. N≤36 [25,26,27], this latter value being the current record, obtained by the length doubling method. The latter group has also determined the current highest values of $c_{N}$ on the BCC (body-centered cubic) (N=28) and FCC (N=24) unrestricted lattices. The continual growth of the range of known values of $c_{N}$ has made it possible to obtain more accurate numerical estimates of the various parameters appearing in Equations (1) and (2). Extrapolation by means of differential approximants and direct fitting to asymptotic expansions yields values for γ and ν in good agreement with those obtained by the MC renormalization group [95], conformal bootstrap [96] and field theory [97].

In this contribution, we present results for the cubic P (SC) and cubic F (FCC) lattices restricted to a pore or “tube” of square cross section. While the complete set {ω} of SAWs on the unrestricted lattice possesses the maximal crystallographic symmetry of space group $Ia\bar{3}d$, the introduction of geometrical restrictions reduces the symmetry on the one hand and, on the other, introduces additional freedom in the definition of the problem. For polymers confined in a pore or tube, the natural correspondence would be to an SAW whose growth is limited in the plane transversal to the tube direction. The new degrees of freedom, which are not meaningful for unrestricted SAWs, are the orientation of the tube axis, the size of its cross-section and the origin of the SAW: the orientation of the tube axis will be defined by direction indices according to crystallographic practice: [ijk]. The cross section will be assumed to be a square of side *L*, measured in units of SAW step length. Finally, $c_{N}$ will be calculated for each distinct origin located on the tube cross section at x=0.

The value of $c_{N}$ will of course depend on the choice of the origin and on the doubly countable infinity of degrees of freedom: direction [ijk] and tube cross section *L*. In the MC simulations of confined polymers that motivate this work, hard-sphere chains confined to tubes of square cross-section are observed to preferentially form quite perfect FCC crystalline domains with their [100] aligned along the tube axis. For both the SC and FCC lattices, we will thus consider the geometrically restricted lattice RL(L) to consist of all the lattice points of coordinates $\underline{x}$ contained in the square-section “tube” defined by:

(3) $$\begin{array}{c} \mathrm{RL}\left(L\right)=\{\underline{x}\;|\;x_{1}\in Z\;,|x_{2}|,|x_{3}|<L\}, \end{array}$$

where the unit of length is the SAW step length. In Equation (3), the tube has been assumed to be oriented parallel to one of the three standard cubic crystallographic axes, or, equivalently, to belong to the direction type 〈100〉. The $x_{1}$ (or *x*) axis [98] has been chosen without loss of generality due to the equivalence of all three axes in the cubic system. The sides of the tube are contained in planes of the crystallographic form {100}.

Unlike in the references cited above, and again motivated by the MC simulations of hard-sphere model polymers confined to tubes, the range of SAW lengths investigated in this work has been kept modest. The reason is twofold: the rich morphological behavior of confined polymers is already clearly observable in MC simulations of comparatively short chains (N≈ 5–15). This can be understood by observing the structural similarity of the ordered chain morphologies presented in the panels of Figure 1 and Figure 2 and which correspond to systems characterized by different chain lengths (from N=7 to 35). Furthermore, once $c_{N}$ in this range is known, it can be used as the basis of reliable approximations for the prediction of entropy-driven phase transitions for much longer chains as well. For these two reasons, we have employed the direct enumeration procedure to determine $c_{N}$.

**Figure 4:**
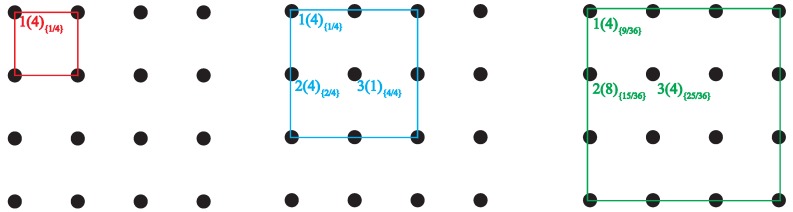
Numbering scheme for all possible origins of SAWs restricted to a tube of square cross section on the cubic P (simple cubic, SC) lattice, for three tube cross section sizes. In all panels, black circles represent lattice points, squares are the tube cross sections: 1×1, 2×2 and 3×3 from left to right. The view is along the tube axis in direction [100]. Numbers on the left correspond to the label of each distinct origin (type). Numbers in parentheses correspond to the cardinality (multiplicity) of each subset. Subindices in braces correspond to area ratios (overlaps), $r^{i}$.

**Figure 5:**
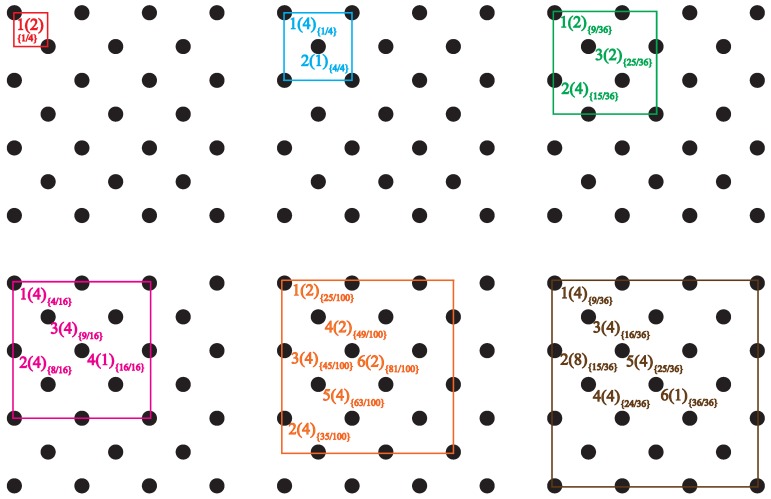
Numbering scheme for all possible origins of SAWs restricted to a tube of square cross section on the cubic F (FCC) lattice, for six tube cross section sizes. In all panels, black circles represent lattice points, squares are the tube cross sections: $0.5\sqrt{2}\times 0.5\sqrt{2}$, $1\sqrt{2}\times 1\sqrt{2}$, $1.5\sqrt{2}\times 1.5\sqrt{2}$, $2\sqrt{2}\times 2\sqrt{2}$, $2.5\sqrt{2}\times 2.5\sqrt{2}$, and $3\sqrt{2}\times 3\sqrt{2}$ from left to right, and top to bottom. The view is along the tube axis in direction [100]. Numbers on the left correspond to the label of each distinct origin (type). Numbers in parentheses correspond to the cardinality (multiplicity) of each subset. Subindices in braces correspond to area ratios (overlaps), $r^{i}$.

The introduction of the tube restriction reduces the symmetry of the full cubic lattice to that of tetragonal space group $I4_{1}/acd$. As a consequence, lattice sites in the tube cross section are not all identical any more, but split into subsets of SAW origins $O_{i}$, all sites in a subset being crystallographically equivalent. We will refer to the cardinality $\left|O_{i}\right|$ of these subsets as their *multiplicity* and will label each of the distinct origins by a *type* which effectively corresponds to the numerical subindex, *i*, of each subset. For example, there are three possible origins for SAWs on an SC lattice restricted by a tube of size 3×3, with multiplicities (type1) $|O_{1}|=4$, (type2) $|O_{2}|=8$, (type3) $|O_{3}|=4$ (Figure 4), and six possible origins for SAWs on an FCC lattice restricted by a tube of size $3\sqrt{2}\times 3\sqrt{2}$, with multiplicities $|O_{1}|=4$, $|O_{2}|=8$, $|O_{3}|=4$, $|O_{4}|=4$, $|O_{5}|=4$ and $|O_{6}|=1$ (Figure 5).

Figure 4 and Figure 5 schematically show the definition of tube size and the numbering/labeling scheme for the SC and FCC restricted lattices, respectively. Thus, an n×n tube has a cross section of the same size as n×n conventional cubic unit cells arranged in a square array, and its side measures L=n units of length (SAW step) for the SC lattice, and $L=n\sqrt{2}$ for the FCC lattice. In these figures, a number placed at selected lattice points is their label, corresponding to the notation *types* in Table A1, Table A2, Table A3, Table A4, Table A5, Table A6, Table A7, Table A8 and Table A9. Each different type corresponds to a different origin for the SAW. The number in parentheses corresponds to the multiplicity of that type (number of crystallographically equivalent restricted lattice points) while the subindex in braces refers to the overlap, to be defined and discussed in Section 4.

As the size of the tube cross section grows, the number of distinct origins (i.e., of different types) increases. The value of $c_{N}$ reported below is given separately for all possible distinct (crystallographically non-equivalent) origins: the values of $c_{N}$ in Table A1, Table A2, Table A3, Table A4, Table A5, Table A6, Table A7, Table A8 and Table A9 correspond to the number of SAWs starting from only one of all equivalent lattice sites of a given type. The value of the multiplicity is a useful piece of information for situations in which the $I4_{1}/acd$ symmetry of the tube is possibly further reduced by other geometrical considerations. For example, a flat, comb-like array of equidistant, identical parallel tubes joined at one end by a common channel loses (among others) all fourfold rotation and screw axes of symmetry, which lowers its space group symmetry to orthorhombic Imma. For the estimation of the entropy of polymers confined to such a nanostructure, origins belonging to the same subset for the isolated tube are, at least in principle, no longer equivalent.

For the calculation of $c_{N}$ for SAWs of the moderate lengths considered in this work, simple enumeration was more than adequate: $c_{N}$ was obtained by exhaustively testing all possible SAWs of length *N* for self-intersections or for violation of the geometrical restrictions, and discarding those that fail to fulfill self-avoidance or geometrical constraint. Computations were carried out on Intel i7-8700K CPUs with 16 GB of memory. For benchmark purposes in the case of unconstrained SAWs, the computational (CPU) time required for the full enumeration of a N=17-SAW in the SC lattice and of a N=13-SAW in the FCC lattice reaches approximately 108 and 928 h, respectively.

It must be emphasized that the goal of this work is not to achieve high-accuracy values [27,85,86,89,99,100] in the calculation of the critical exponents or the leading or sub-leading correction-to-scaling exponents, but to obtain correlations for $c_{N}$ for chains of moderate length to be used in the understanding of the entropic mechanisms of phase transitions observed in the off-lattice (continuum) simulations of confined and densely-packed polymers.

## 3. Results

The values of $c_{N}$ for SAWs on lattices restricted to a tube of cross section L×L oriented along the 〈100〉 direction are presented in Table A1, Table A2 and Table A3 for the SC lattice, together with their average squared end-to-end distance. The corresponding results for the FCC lattice can be found in Table A4, Table A5, Table A6, Table A7, Table A8 and Table A9. SAW origin types correspond to the labeling schemes of Figure 4 and Figure 5. The coefficients of best fit of the scaling laws in Equations (1) and (2) to the data of Table A1, Table A2, Table A3, Table A4, Table A5, Table A6, Table A7, Table A8 and Table A9 are shown in Table 1 and Table 2. As expected, the values of all coefficients are specific for each lattice type, tube size and type of origin. Within a given tube size, restricted SAWs starting at more confined lattice sites (lower type) have systematically lower values of $c_{N}$ than those further removed from the boundaries. Thus, for SAWs of N=17 restricted to a 3×3 tube in the SC lattice, $c_{N}=9,239,393,494$ for the more confined, in the corner of the tube, type 1 (of multiplicity 4), $c_{N}=12,003,817,994$ for the less confined type 2 (on the side wall with multiplicity 8) and $c_{N}=14,972,474,238$ for the least confined type 3 (with multiplicity 4). For comparison, using the same number of steps the number of different SAW configurations is (N=17) $c_{N}=473,730,252,102$ for the unrestricted SC lattice.

**Figure 6:**
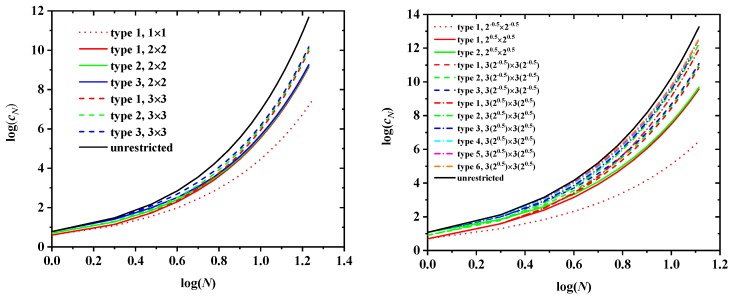
Log-log plot of the number of distinct SAW configurations, $c_{N}$, versus the number of SAW steps, *N*, for the SC (left panel) and the FCC (right panel) lattices. Tube cross-sections correspond to 1×1, 2×2 and 3×3 for SC and to $0.5\sqrt{2}\times 0.5\sqrt{2}$, $1\sqrt{2}\times 1\sqrt{2}$, $1.5\sqrt{2}\times 1.5\sqrt{2}$ and $3\sqrt{2}\times 3\sqrt{2}$ for FCC. For a given lattice and confining tube, results are shown for every possible distinct SAW origin (type). Also shown for comparison are the corresponding curves for the unrestricted lattices (solid black lines).

Based on the results presented in Table A1, Table A2, Table A3, Table A4, Table A5, Table A6, Table A7, Table A8 and Table A9 Figure 6 shows the log-log plot of the number of distinct SAWs, $c_{N}$, versus the number of SAW steps, *N*, for all SC (left panel) and selected FCC (right panel) lattices for different SAW origins (types) and sizes of the confining tube. Also shown for comparison purposes are the corresponding results for the unrestricted cases. It can be clearly seen that, for a given tube size the closer to the tube surface, the lower the total number of distinct SAWS; for origin types residing in the corner of the tube, the larger the tube size, the larger the SAW population. Compared to the unrestricted case, type 1 (corner) of the smallest tube shows always the largest difference while the type of highest value (farthest from the corner) of the largest tube shows the closest similarity, independently of lattice type.

**Figure 7:**
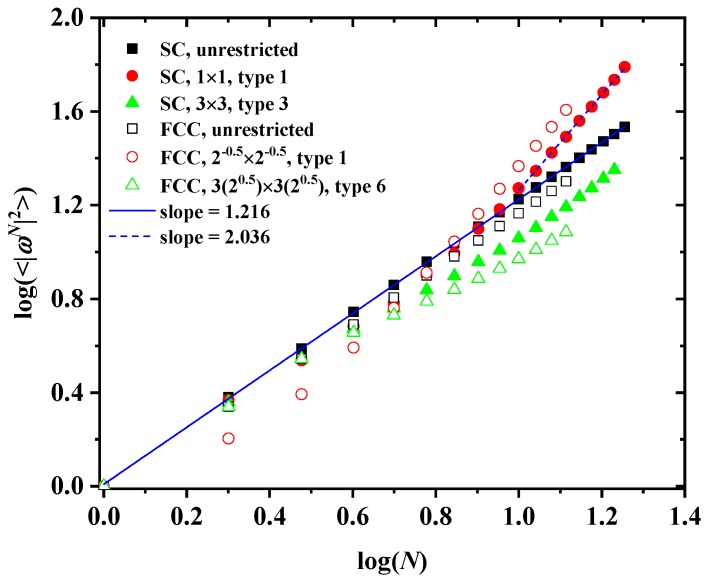
Log-log plot of the average squared end-to-end distance, $\langle {\left|\omega^{N}\right|}^{2}\rangle$, versus the number of SAW steps, *N*, for the SC (filled symbols) and the FCC (open symbols) lattices. The black color corresponds to unrestricted lattices, while red and green to confined ones. The solid blue line corresponds to best linear fit on the whole range of SAW data for unrestricted SC lattice. The dashed blue line corresponds to best linear fit on the late-*N* SAW range for the most confined SC case (type 1 in 1×1 tube).

We should note here that Equation (1), quantifying the dependence of $c_{N}$ on *N*, is manifestly valid for the whole range of studied systems, independently of lattice type, tube confinement and SAW origin. However, the same is not true for Equation (2) which relates SAW size, as quantified by the average square end-to-end distance, with a number of SAW steps. For the unrestricted lattice, Equation (2) remains accurate in the whole *N*-range. In sharp contrast, for the confined lattices, especially for SAW origins near the confining tube, anomalous behavior is clearly observed for small-*N* SAWs. This is particularly evident in the results of Figure 7 showing log-log plots of $\langle {\left|\omega^{N}\right|}^{2}\rangle$ versus *N* for SC (filled symbols) and FCC (open symbols) unrestricted (black color) and confined (red or green color) lattices. For the latter, we differentiate between SAW origins corresponding to the most (SC: type 1 in 1×1 tube; FCC: type 1 in $0.5\sqrt{2}\times 0.5\sqrt{2}$) and least (SC: type 3 in 3×3 tube; FCC: type 6 in $3\sqrt{2}\times 3\sqrt{2}$) confined cases. The combination of spatial restrictions along with the anisotropy in cell size leads to this anomalous scaling for early-*N* SAWs. Thus, all *D* and ν coefficients reported in Table 1 and Table 2 correspond to fittings applied on data covering the late-*N* SAW regime.

**Figure 8:**
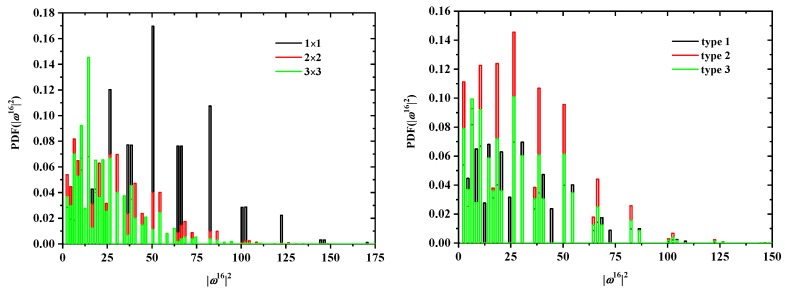
Probability distribution function for ${\left|\omega^{16}\right|}^{2}$ for SAWs of fixed length N=16 on restricted SC lattices. The left panel shows the effect of the tube cross section for a fixed SAW origin (type 1); the right panel depicts the effect of SAW origin (type) for a fixed tube cross section (2×2).

**Figure 9:**
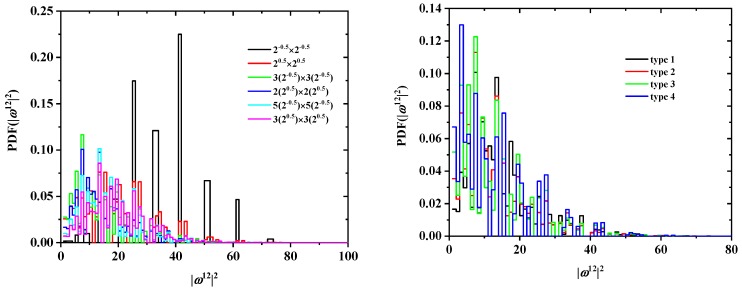
Probability distribution function for ${\left|\omega^{12}\right|}^{2}$ for SAWs of fixed length N=12 on restricted FCC lattices. The left panel shows the effect of tube cross section for a fixed SAW origin (type 1); the right panel depicts the effect of SAW origin (type) for a fixed tube cross section ($2\sqrt{2}\times 2\sqrt{2}$).

**Figure 10:**
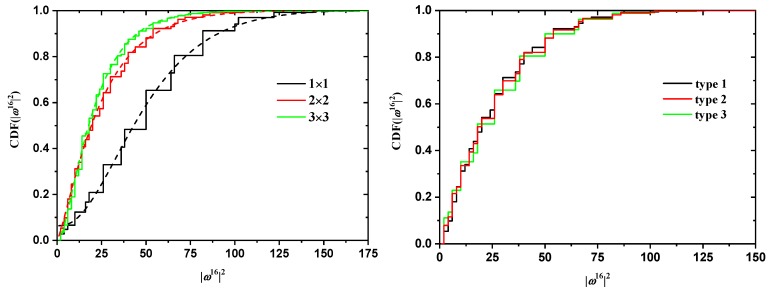
Cumulative probabilities for the distribution functions of ${\left|\omega^{16}\right|}^{2}$ for SAWs of fixed length N=16 on restricted SC lattices of Figure 8. Also shown in the left panel are best fits using the gamma function.

**Figure 11:**
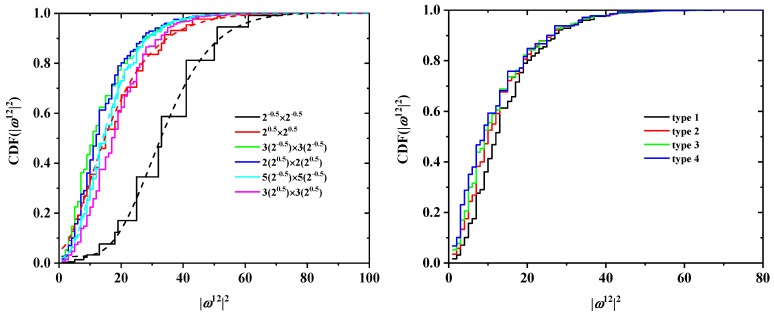
Cumulative probabilities for the distribution functions of ${\left|\omega^{12}\right|}^{2}$ for SAWs of fixed length N=12 on restricted FCC lattices of Figure 9. Also shown in the left panel are best fits using the gamma function for selected cases.

In addition to $c_{N}$ and $\langle {\left|\omega^{N}\right|}^{2}\rangle$, the discrete probability distribution functions (PDF) and cumulative distribution functions (CDF) of ${\left|\omega^{N}\right|}^{2}$ are available. In Figure 8 and Figure 9, the effects of tube size (left panel), for a fixed SAW origin, and of origin type (right panel), for a fixed tube cross section, on the distribution for SAWs of length N=16 are presented for the SC and FCC lattices, respectively. As expected, higher confinement (i.e., smaller tube cross section) leads to more stretched SAWs and a distribution shifted to higher values of ${\left|\omega^{16}\right|}^{2}$ (remarkably higher histogram values above ${\left|\omega^{16}\right|}^{2}$ at and above 50). This shift is particularly evident in the cumulative distributions (left panels of Figure 10 and Figure 11). The strong confinement induced by the small tube 1×1 definitely leads to significantly more stretched SAWs.

On the other hand, the SAW origin type has little influence on the spread of the distribution, but it does increase or reduce the probability of certain SAW extensions (see, for example, the higher red bars in the right panel of Figure 8). It is also remarkable that, for a given *N* and tube cross section, the most confined SAWs (type 1 in this case) show non-vanishing probabilities for values of ${\left|\omega^{16}\right|}^{2}$ for which the probability for types 2 and 3 is zero (isolated black bars in the plot of Figure 8 at ${\left|\omega^{16}\right|}^{2}=12,24,44,73$). Identical conclusions can be drawn for the effect of origin type and tube length for SAWS on FCC lattices according to the probability distributions presented in Figure 9. As can be seen in the right panels of Figure 10 and Figure 11, there is virtually no difference in the cumulative distributions for the different types of SAW origins.

**Figure 12:**
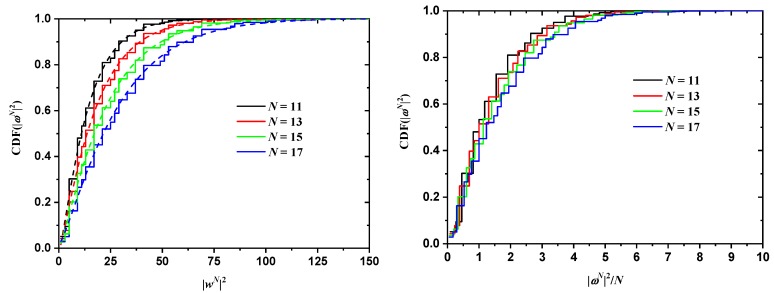
Cumulative probability distribution function for ${\left|\omega^{N}\right|}^{2}$ for SAWs of different length in a 2×2 tube and for SAW origin of type 1 (left panel) on restricted SC lattices. The right panel shows the same distributions, scaled by 1/N, which for a step length of 1 is numerically equivalent to the characteristic ratio of the SAW [101]. Also shown in the left panel are best fits using the gamma function.

**Figure 13:**
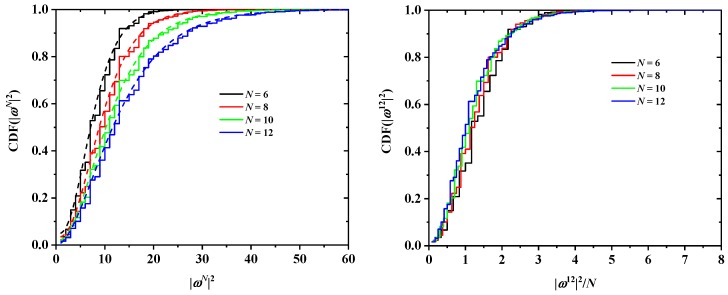
Cumulative probability distribution function for ${\left|\omega^{N}\right|}^{2}$ for SAWs of different length in a $2\sqrt{2}\times 2\sqrt{2}$ tube and for SAW origin of type 1 (left panel) on restricted FCC lattices. The right panel shows the same distributions, scaled by 1/N, which for a step length of 1 is numerically equivalent to the characteristic ratio of the SAW [101]. Also shown in the left panel are best fits using the gamma function.

The effect of chain length on the cumulative distribution of ${\left|\omega^{N}\right|}^{2}$ is shown in Figure 12 and Figure 13 for the SC and FCC lattices, respectively. With respect to SC, according to the data in Figure 12, the four curves corresponding to N=11,13,15,17 (left panel) are noticeably different, as they should be for different values of *N*. However, they come much closer together when scaled by 1/N (right panel of the same figure). In other words, the characteristic ratio of the SAWs is fairly constant in this range of *N*, with a median value of approx. 1.25. A very similar conclusion can be drawn for the FCC case (Figure 13), where the characteristic ratio shows little variation with the number of SAW steps.

## 4. Discussion

An inspection of the tables shows that $c_{N}$ is, as expected, lower for the restricted lattices than for the unrestricted ones, the more so, the smaller the restricting tube. The black, solid line in both panels of Figure 6 represents in log-log scale the growth of $c_{N}$ with SAW length *N* for the unrestricted case, while all other lines correspond to the value of $c_{N}$ for SAWs restricted on confining tubes of specific sizes for all possible different origins, both on the cubic P (left panel) and F (right panel) lattices.

The faster growth of $c_{N}$ for unrestricted SAWs is also reflected in the larger values of the connective constant μ, which is the dominant term in Equation (1) for large values of *N*: $\mu^{SC}=4.719$ for the unrestricted SC lattice, against $\mu_{r}^{SC}=3.798$ (multiplicity-based, weighted average over all three types of origin) for the restricted 3 × 3 SC lattice, while the corresponding value drops to just $\mu^{SC}=2.410$ for the 1×1 tube, a decrease of approximately 50% with respect to the bulk case. For the FCC lattice, the analogous numbers are: $\mu^{FCC}=10.06$ (unrestricted), $\mu_{r}^{FCC}=8.751$ (weighted average over all six types of origin for the restricted $3\sqrt{2}\times 3\sqrt{2}$ FCC lattice) and $\mu^{FCC}=2.674$ for the most confined $0.5\sqrt{2}\times 0.5\sqrt{2}$ FCC case, the latter being around 75% less than the value of the unrestricted FCC SAW. This behavior is in agreement with the geometrical meaning of connectivity: restricted SAWs that start close to one of the boundaries have, on average, fewer neighbors than those that start farther from the confining tube.

In addition, the average (weighted by the type multiplicity) connectivity constants in Table 1 and Table 2 reflect this trend very clearly: as tube size increases, the values of the average connectivity constant increase and approach the unrestricted values. For FCC lattices of sizes $0.5\sqrt{2}\times 0.5\sqrt{2}$, $1\sqrt{2}\times 1\sqrt{2}$, $1.5\sqrt{2}\times 1.5\sqrt{2}$, $2\sqrt{2}\times 2\sqrt{2}$, $2.5\sqrt{2}\times 2.5\sqrt{2}$ and $3\sqrt{2}\times 3\sqrt{2}$, the multiplicity-weighted average values of μ are 2.674 (73.4%), 4.742 (52.9%), 6.491 (35.5%), 7.613 (24.3%), 8.344 (17.2%) and 8.751 (13.1%), where numbers in parentheses denote percentage reduction with respect to the connectivity constant of the bulk FCC lattice.

Furthermore, for a given size of the tube, the values of $c_{N}$ for different origins tend to converge as *N* grows. This is most clearly observed in the left panel of Figure 6: the curves for the three origin types are already quite close for the moderate value N=17 for all restricted SC lattices. The same is true for the SAWs of length N=12 on confined FCC lattices as seen in the right panel of Figure 6. For a given lattice type (FCC or SC) and a given spatial restriction (tube cross section), the value of $c_{N}$ must approach a common limit as N→∞, independently of the particular type of SAW origin: sufficiently long SAWs lose the “memory” of their starting point so that:

$$\begin{array}{c} \lim_{N\to \infty }\frac{\log c_{N}^{i}}{\log c_{N}^{j}}=1\;i\in O_{i},\;j\in O_{j}\;i\neq j \end{array}$$

must hold, where $O_{k}$ is one of the sets of equivalent SAW origins for a restricted lattice, and $c_{N}^{i}$ is the number of restricted SAWs of length *N* starting at an origin of type $i\in O_{i}$. The rate at which $c_{N}^{i}$ approaches this common N→∞ limit is of course dependent on the lattice. As can be seen in Figure 6, SAWs on the restricted FCC lattice tend to this limit more slowly than SAWs on the SC one.

**Figure 14:**
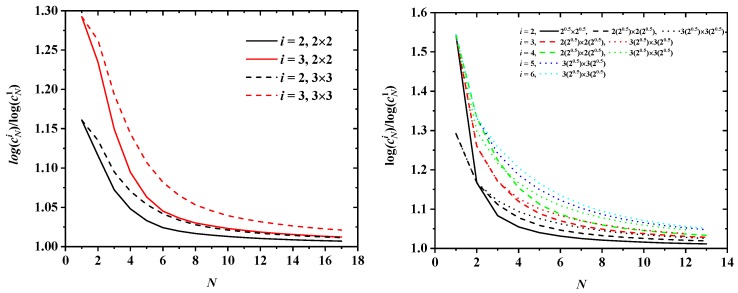
Ratio $\frac{\log c_{N}^{i}}{\log c_{N}^{1}}$ as a function of SAW steps, *N*, for different SAW origins $i=2,\ldots ,|O_{k}|$ on (**left**): 2×2 (solid lines) and 3×3 (dashed lines) SC and (**right**): $1\sqrt{2}\times 1\sqrt{2}$ (solid line), $2\sqrt{2}\times 2\sqrt{2}$ (dashed lines) and $3\sqrt{2}\times 3\sqrt{2}$ (dotted lines) FCC lattices.

In Figure 14, the ratio $\frac{\log c_{N}^{i}}{\log c_{N}^{1}}$ for different SAW origins (i.e., the ratio of the curves represented in Figure 6 divided by the curve for $c_{N}$ of SAW origin of type 1, taken arbitrarily as reference) is seen to indeed approach unity as *N* increases for both SC (left panel) and FCC (right panel) lattices. Systematically, the ratio tends faster to unity for SAW origins that lie close in space and for smaller tube cross sections. For example, for an SAW of length N=13 on the $3\sqrt{2}\times 3\sqrt{2}$ FCC lattice for type of origin i=2, 4 and 6, the corresponding ratios are 1.026, 1.048 and 1.051. In parallel, for an SAW of N=17 steps on a SC lattice with origin type 2, the ratio increases from 1.007 for a 2×2 tube to 1.011 for a 3×3 one.

**Figure 15:**
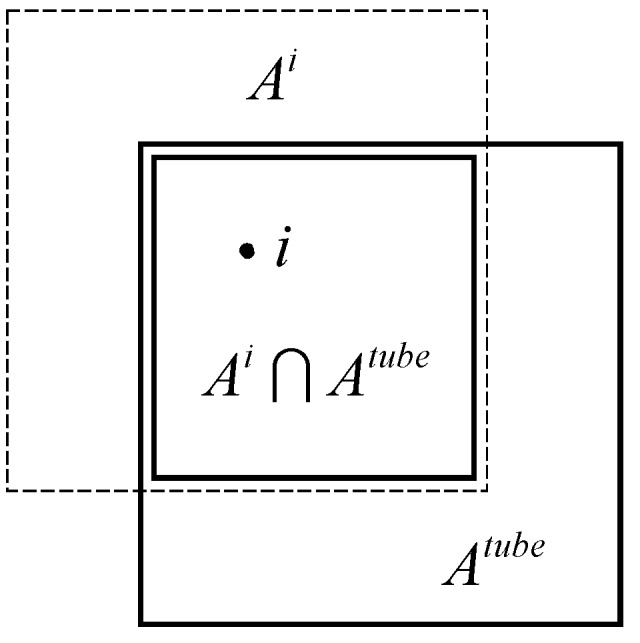
The overlap $r^{i}$ is defined as the area (small square) common to a tube cross section centered at the origin of type *i* (dashed line) and the tube cross section (solid line), divided by the complete tube cross section.

The dependence of $c_{N}$ on SAW origin (type) for given *N* and tube size can be explained, at least approximately, by a simple geometric argument. Since a higher degree of confinement leads to a greater reduction in $c_{N}$, it seems natural to attempt a scaling of $c_{N}^{i}$ by means of the following area ratio or *overlap*:

$$\begin{array}{c} r^{i}=\frac{a(A^{i}\cap A^{tube})}{a(A^{tube})}\leq 1, \end{array}$$

where $a(A^{i}\cap A^{tube})$ is the area common to a tube cross section (a square in the present work) centered at the SAW origin of type *i* (square in dotted line in Figure 15), and the tube cross section. The overlap $r^{i}$ is the ratio of this area (small square in Figure 15) to the entire tube cross section. More highly confined SAW origins (i.e., a corner, like type 1 in the 3×3 restricted SC lattice) have lower values of $r^{i}$, while those close to the center of the tube have higher $r^{i}$. Taking the SC lattice restricted by a 3×3 tube (rightmost panel in Figure 4) as an illustrative example, the values of the overlap for the three distinct types of origin are:

$$\begin{array}{c} r^{1}=\frac{1}{4}\;r^{2}=\frac{5}{12}\;r^{3}=\frac{25}{36}. \end{array}$$

The overlap values for all SAW origin types in the SC and FCC lattices used in the present work are reported in braces in the schemes of Figure 4 and Figure 5. In fact, going back to the sketches, the labeling of the distinct types of SAW origins is based on the overlap value of a given site: the lower the overlap value, the lower the origin index. According to the definition, overlap values for the SC and FCC lattices, confined in tube with direction type 〈100〉, are bounded between 0.25 (assigned always to origin type 1) and 1. As can be seen in the reported area ratios of Figure 4 and Figure 5 for a given tube size, no two distinct origin types have the same overlap value. With respect to the confined $3\sqrt{2}\times 3\sqrt{2}$ FCC lattice, origin types 1, 2, 3, 4, 5 and 6 are characterized by area ratios (overlaps) of 9/36, 15/36, 16/36, 24/36, 25/36 and 36/36, respectively.

**Figure 16:**
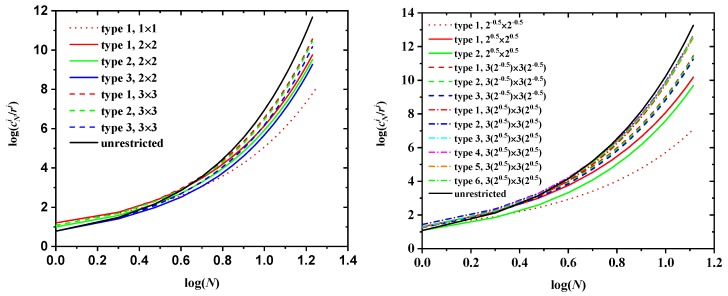
Log-log plot of the number of distinct SAW configurations scaled by the inverse overlap, $c_{N}^{i}/r^{i}$, as a function of SAW steps, *N*, on confined SC (left panel) and FCC lattices (right panel) for various origin types and tube cross-sections. Also shown for comparison are the corresponding results for the unrestricted SAW (solid black line).

Based on the above, it is tempting to study the behavior of the curves $\frac{c_{N}^{i}}{r^{i}}$ (log-log plots in Figure 16) versus *N*, where now the number of distinct SAW configurations for a given origin type is divided by the corresponding overlap of that type. The comparison of the left panel of Figure 6 with Figure 16 strongly suggests that this simple geometric argument does indeed successfully explain to first order the dependence of $c_{N}$ on the type of SAW origin. Curves corresponding to different tube cross-sections and origin types seem to be brought closer together when they are scaled by the proper overlap values.

## 5. Conclusions

In this paper, we have evaluated the number and characteristic dimensions of SAWs of moderate length on the simple and face centered cubic lattices restricted to a tube of square cross section oriented along crystallographic directions of the type <100>. Both the number of restricted SAWs and their average size (given by the average squared end-to-end distance) have also been fitted to the functional form of scaling laws for unrestricted SAWs. This knowledge is an essential requirement for the calculation of the entropy in dense packings of hard-sphere chains in restricted geometries and thus for the understanding and prediction of their phase transitions under conditions of extreme confinement. For example, according to the modeling work of Ref. [54], ordered morphologies of defective, coexisting FCC crystals of different orientations are spontaneously formed in dense packings of hard-sphere chains confined in a square tube. Such crystal morphologies are connected through structural transitions driven solely by entropy.

## Figures and Tables

**Table 1:** Calculated coefficients in scaling laws (Equations (1) and (2)) for SC lattice restricted to a tube oriented along [100]. Universal exponents for unrestricted SAWs are marked with an asterisk *.

Tube Size	Type	*A*	μ	γ	*D*	ν
1 × 1	1	1.634	2.410	1.417	0.151	1.039
2 × 2	1	1.171	3.354	1.202	0.399	0.750
	2	1.519	3.262	1.289	0.315	0.794
	3	1.926	3.133	1.430	0.259	0.834
3 × 3	1	0.993	3.975	0.923	1.610	0.477
	2	1.303	3.806	1.133	1.052	0.543
	3	1.661	3.606	1.393	0.656	0.620
unrestricted SC lattice:		1.269	4.719	1.102 *	1.046	0.603 *

**Table 2:** Calculated coefficients in scaling laws (1) and (2) for FCC lattice restricted to a tube oriented along [100]. Universal exponents for unrestricted SAWs are marked with an asterisk *.

Tube Size	Type	*A*	μ	γ	*D*	ν
$0.5\sqrt{2}\times 0.5\sqrt{2}$	1	1.876	2.674	1.564	0.187	1.047
$1\sqrt{2}\times 1\sqrt{2}$	1	1.063	4.696	1.745	0.203	0.899
	2	2.430	4.928	1.296	0.171	0.952
$1.5\sqrt{2}\times 1.5\sqrt{2}$	1	0.747	6.615	1.352	0.710	0.597
	2	1.213	6.540	1.331	0.477	0.671
	3	1.917	6.267	1.410	0.314	0.756
$2\sqrt{2}\times 2\sqrt{2}$	1	0.622	7.987	1.030	1.914	0.404
	2	1.062	7.512	1.282	1.163	0.480
	3	1.586	7.532	1.207	0.910	0.520
	4	1.764	6.843	1.634	0.521	0.624
$2.5\sqrt{2}\times 2.5\sqrt{2}$	1	0.568	8.790	0.844	2.420	0.384
	2	0.911	8.740	0.873	1.916	0.408
	3	0.957	8.347	1.128	1.687	0.421
	4	1.413	8.477	1.004	1.421	0.444
	5	1.494	8.023	1.279	1.182	0.467
	6	1.577	7.606	1.544	0.910	0.505
$3\sqrt{2}\times 3\sqrt{2}$	1	0.544	9.200	0.749	2.515	0.403
	2	0.906	8.827	1.028	1.849	0.425
	3	1.335	8.995	0.889	1.578	0.448
	4	1.396	8.575	1.200	1.318	0.460
	5	1.460	8.224	1.415	1.262	0.454
	6	1.456	8.172	1.505	1.062	0.474
unrestricted cubic F lattice:		1.190	10.06	1.135 *	0.934	0.598 *

## Abbreviations

The following abbreviations are used in this manuscript:

FCC	Face Centered Cubic
MC	Monte Carlo
MD	Molecular Dynamics
SAW	Self-Avoiding Walk
SC	Simple Cubic
CCE	Characteristic Crystallographic Element (norm)
BCC	Body Centered Cubic
PDF	Probability Distribution Function
CDF	Cumulative Distribution Function

## Appendix A

**Table A1:** SC lattice, tube cross section 1.0 × 1.0. The second column of the first table is the value of *c_N_* for SAWs on the unrestricted SC lattice, included for comparison purposes.

	Type 1	Multiplicity |*O*_1_| = 4	
*N*	*c_N_* Unrestricted	*c_N_*	$\langle {\left|\omega^{N}\right|}^{2}\rangle$
1	6	4	1.000
2	30	12	2.333
3	150	36	3.444
4	726	98	4.816
5	3534	274	6.051
6	16,926	702	7.977
7	81,390	1854	9.846
8	387,966	4614	12.56
9	1,853,886	11,778	15.20
10	8,809,878	28,914	18.73
11	41,934,150	72,394	22.19
12	198,842,742	176,310	26.59
13	943,974,510	435,346	30.98
14	4,468,911,678	1,055,730	36.29
15	21,175,146,054	2,584,026	41.66
16	100,121,875,974	6,249,358	47.94
17	473,730,252,102	15,208,438	54.34
18	2 237,723,684,094	36,724,294	61.60

**Table A2:** SC lattice, tube cross section 2.0 × 2.0.

	Type 1	Multiplicity |*O*_1_| = 4		Type 2	Multiplicity |*O*_2_| = 4		Type 3	Multiplicity |*O*_3_| = 1
*N*	*c_N_*	$\langle {\left|\omega^{N}\right|}^{2}\rangle$	*N*	*c_N_*	$\langle {\left|\omega^{N}\right|}^{2}\rangle$	*N*	*c_N_*	$\langle {\left|\omega^{N}\right|}^{2}\rangle$
1	4	1.000	1	5	1.000	1	6	1.000
2	14	2.571	2	19	2.316	2	26	2.154
3	54	3.963	3	72	3.556	3	98	3.122
4	200	5.420	4	258	4.853	4	330	4.170
5	744	6.634	5	926	5.916	5	1130	5.120
6	2626	7.925	6	3 176	7.146	6	3746	6.388
7	9186	9.051	7	11,000	8.276	7	12,802	7.581
8	31,122	10.37	8	36 988	9.670	8	42,498	9.120
9	105,766	11.63	9	125,302	11.01	9	143,610	10.58
10	351,798	13.18	10	414,518	12.68	10	472,242	12.42
11	1,175,726	14.71	11	1,381,390	14.31	11	1 570,714	14.19
12	3 859,350	16.59	12	4 515,022	16.31	12	5 110,426	16.36
13	12,729,142	18.46	13	14,853,462	18.30	13	16,779,354	18.46
14	41,355,642	20.71	14	48,105,654	20.67	14	54,148,874	21.00
15	134,970,238	22.96	15	156,694,796	23.03	15	176,058,234	23.49
16	435,124,318	25.60	16	504,010,840	25.80	16	564,679,330	26.43
17	1,408,619,206	28.25	17	1,629,120,330	28.56	17	1,822,489,530	29.34

**Table A3:** SC lattice, tube cross section 3.0 × 3.0.

	Type 1	Multiplicity |*O*_1_| = 4		Type 2	Multiplicity |*O*_2_| = 8		Type 3	Multiplicity |*O*_3_| = 4
*N*	*c_N_*	$\langle {\left|\omega^{N}\right|}^{2}\rangle$	*N*	*c_N_*	$\langle {\left|\omega^{N}\right|}^{2}\rangle$	*N*	*c_N_*	$\langle {\left|\omega^{N}\right|}^{2}\rangle$
1	4	1.000	1	5	1.000	1	6	1.000
2	14	2.571	2	20	2.400	2	28	2.286
3	56	4.143	3	82	3.780	3	122	3.492
4	224	5.911	4	328	5.311	4	488	4.721
5	926	7.505	5	1336	6.683	5	1926	5.760
6	3738	9.179	6	5273	8.107	6	7328	6.885
7	15,056	10.64	7	20,813	9.331	7	28,132	7.896
8	59,092	12.09	8	80,282	10.61	8	106,004	9.068
9	230,254	13.36	9	309,654	11.76	9	403,470	10.17
10	881,850	14.65	10	1,175,480	13.02	10	1,512,774	11.46
11	3,367,124	15.84	11	4 466,712	14.20	11	5 715 168	12.70
12	12,712,194	17.13	12	16,770,216	15.54	12	21,299,430	14.15
13	47,952,018	18.38	13	63,066,644	16.85	13	79,832,758	15.55
14	179,317,400	19.77	14	234,827,439	18.33	14	295,630,770	17.18
15	670,507,498	21.17	15	875,986,779	19.80	15	1,099,932,734	18.77
16	2,488,658,374	22.73	16	3,239,657,890	21.47	16	4,049,793,742	20.60
17	9,239,393,494	24.31	17	12,003,817,994	23.13	17	14,972,474,238	22.38

**Table A4:** FCC lattice, tube cross section $0.5\sqrt{2}\times 0.5\sqrt{2}$. The second column of the first table is the value of $c_{N}$ for SAWs on the unrestricted FCC lattice, included for comparison purposes.

	Type 1	Multiplicity |*O*_1_| = 2	
*N*	*c_N_* Unrestricted	*c_N_*	$\langle {\left|\omega^{N}\right|}^{2}\rangle$
1	12	5	1.000
2	132	20	1.600
3	1404	68	2.471
4	14,700	208	3.904
5	152,532	624	5.776
6	1,573,716	1840	8.157
7	16,172,148	5360	11.07
8	165,697,044	15,488	14.56
9	1,693,773,924	44,608	18.61
10	17,281,929,564	128,192	23.22
11	176,064,704,412	368,064	28.39
12	1,791,455,071,068	1,056,000	34.13
13	18,208,650,297,396	3,028,992	40.43

**Table A5:** FCC lattice, tube cross section $1.0\sqrt{2}\times 1.0\sqrt{2}$.

	Type 1	Multiplicity |*O*_1_| = 4		Type 2	Multiplicity |*O*_2_| = 4
*N*	*c_N_*	$\langle {\left|\omega^{N}\right|}^{2}\rangle$	*N*	*c_N_*	$\langle {\left|\omega^{N}\right|}^{2}\rangle$
1	5	1.000	1	12	1.000
2	39	2.256	2	72	1.556
3	248	3.113	3	392	2.265
4	1460	3.907	4	2176	3.199
5	8132	4.756	5	11,680	4.286
6	43,860	5.816	6	61,136	5.633
7	230,476	7.106	7	314,416	7.226
8	1,190,588	8.657	8	1,600,960	9.073
9	6,072,572	10.47	9	8,070,448	11.20
10	30,677,292	12.57	10	40,350,672	13.63
11	153,744,188	14.97	11	200,495,840	16.38
12	765,753,696	17.68	12	992,030,176	19.45
13	3,796,189,560	20.70	13	4 893,578,576	22.85

**Table A6:** FCC lattice, tube cross section $1.5\sqrt{2}\times 1.5\sqrt{2}$.

	Type 1	Multiplicity |*O*_1_| = 2		Type 2	Multiplicity |*O*_2_| = 4		Type 3	Multiplicity |*O*_3_| = 2
*N*	*c_N_*	$\langle {\left|\omega^{N}\right|}^{2}\rangle$	*N*	*c_N_*	$\langle {\left|\omega^{N}\right|}^{2}\rangle$	*N*	*c_N_*	$\langle {\left|\omega^{N}\right|}^{2}\rangle$
1	5	1.000	1	8	1.000	1	12	1.000
2	39	2.256	2	62	2.097	2	101	1.941
3	317	3.738	3	487	3.234	3	736	2.707
4	2456	4.927	4	3643	4.223	4	5152	3.468
5	18,028	5.920	5	26,106	5.096	5	35,522	4.299
6	127,242	6.813	6	181,783	5.960	6	241,888	5.216
7	876,392	7.705	7	1,240,790	6.878	7	1,627,468	6.236
8	5,934,196	8.661	8	8,342,670	7.894	8	10,825,480	7.377
9	39,648,964	9.725	9	55,415,928	9.034	9	71,271,844	8.656
10	261,993,600	10.92	10	364,364,782	10.32	10	465,099,616	10.08
11	1,715,097,328	12.27	11	2,375,202,602	11.76	11	3,012,465,424	11.67
12	11,139,357,984	13.79	12	15,371,509,668	13.36	12	19,389,036,972	13.43
13	71,869,479,512	15.47	13	98,873,697,150	15.14	13	124,130,404,052	15.36

**Table A7:** FCC lattice, tube cross section $2.0\sqrt{2}\times 2.0\sqrt{2}$.

	**Type 1**	**Multiplicity |*O*_1_| = 4**		**Type 2**	**Multiplicity |*O*_2_| = 4**
***N***	***c_N_***	$\langle {\left|\omega^{N}\right|}^{2}\rangle$	***N***	***c_N_***	$\langle {\left|\omega^{N}\right|}^{2}\rangle$
1	5	1.000	1	8	1.000
2	39	2.256	2	72	2.222
3	317	3.738	3	602	3.326
4	2707	5.402	4	5018	4.556
5	22,778	6.887	5	41,050	5.692
6	186,798	8.169	6	328,378	6.703
7	1,493,410	9.278	7	2 577,480	7.640
8	11,705,520	10.28	8	19,944,688	8.557
9	90,414,004	11.23	9	152,636,704	9.491
10	690,737,504	12.19	10	1 157,776,248	10.47
11	5,231,407,492	13.18	11	8,716,517,832	11.52
12	39,334,158,792	14.23	12	65,200,437,688	12.65
13	293,889,553,284	15.37	13	484,934,433,160	13.88
	**Type 3**	**Multiplicity |*O*_3_| = 4**		**Type 4**	**Multiplicity |*O*_4_| = 1**
***N***	***c_N_***	$\langle {\left|\omega^{N}\right|}^{2}\rangle$	***N***	***c_N_***	$\langle {\left|\omega^{N}\right|}^{2}\rangle$
1	12	1.000	1	12	1.000
2	101	1.941	2	132	2.182
3	847	3.116	3	1152	2.958
4	6946	4.152	4	9144	3.636
5	55,498	5.088	5	70,400	4.353
6	435,926	5.985	6	536,376	5.144
7	3,379,684	6.879	7	4,071,072	6.012
8	25,926,400	7.797	8	30,796,856	6.961
9	197,133,924	8.763	9	231,952,920	7.991
10	1,487,560,076	9.795	10	1,738,210,872	9.107
11	11,150,268,460	10.91	11	12,958,623,176	10.31
12	83,085,654,372	12.11	12	96,129,954,888	11.61
13	615,859,395,980	13.41	13	709,838,117,576	13.02

**Table A8:** FCC lattice, tube cross section $2.5\sqrt{2}\times 2.5\sqrt{2}$.

	**Type 1**	**Multiplicity |*O*_1_| = 2**		**Type 2**	**Multiplicity |*O*_2_| = 4**
***N***	***c_N_***	$\langle {\left|\omega^{N}\right|}^{2}\rangle$	***N***	***c_N_***	$\langle {\left|\omega^{N}\right|}^{2}\rangle$
1	5	1.000	1	8	1.000
2	39	2.256	2	62	2.097
3	317	3.738	3	522	3.421
4	2707	5.402	4	4508	4.922
5	23,701	7.209	5	39,468	6.465
6	208,144	8.941	6	344,215	7.922
7	1,810,302	10.50	7	2 966 304	9.241
8	15,526,912	11.89	8	25,216,726	10.43
9	131,356,780	13.18	9	211,725,485	11.52
10	1,098,163,378	14.24	10	1,759,351 811	12.54
11	9,092,485,480	15.28	11	14,497,192,414	13.54
12	74,701,087,430	16.29	12	118,646,116,612	14.52
13	609,855,297,956	17.29	13	965,528,829,603	15.53
	**Type 3**	**Multiplicity |*O*_3_| = 4**		**Type 4**	**Multiplicity |*O*_4_| = 2**
***N***	***c_N_***	$\langle {\left|\omega^{N}\right|}^{2}\rangle$	***N***	***c_N_***	$\langle {\left|\omega^{N}\right|}^{2}\rangle$
1	8	1.000	1	12	1.000
2	72	2.222	2	101	1.941
3	637	3.474	3	847	3.116
4	5557	4.763	4	7365	4.472
5	48,366	6.108	5	63,980	5.751
6	418,016	7.410	6	549,602	6.915
7	3,570,910	8.604	7	4,663,884	7.987
8	30,133,676	9.693	8	39,130,524	8.997
9	251,551,004	10.71	9	325,115,970	9.971
10	2,081,126,958	11.69	10	2,679,470,380	10.93
11	17,091,369,920	12.66	11	21,936,104,286	11.90
12	139,509,610,898	13.64	12	178,579,440,256	12.90
13	1,132,860,537,091	14.66	13	1,446,780,259,612	13.94
	**Type 5**	**Multiplicity |*O*_5_| = 4**		**Type 6**	**Multiplicity |*O*_6_| = 2**
***N***	***c_N_***	$\langle {\left|\omega^{N}\right|}^{2}\rangle$	***N***	***c_N_***	$\langle {\left|\omega^{N}\right|}^{2}\rangle$
1	12	1.000	1	12	1.000
2	116	2.069	2	132	2.182
3	1044	3.176	3	1277	3.249
4	9138	4.292	4	11,348	4.143
5	78,471	5.355	5	96,462	4.951
6	664,057	6.347	6	802,244	5.743
7	5,558,369	7.293	7	6,601,488	6.553
8	46,127,001	8.218	8	54,022,204	7.400
9	380,120,277	9.144	9	440,478,598	8.292
10	3,113,966,985	10.09	10	3 580,119,048	9.236
11	25,377 886,728	11.06	11	29,005,342,540	10.24
12	205,863,958,205	12.08	12	234,222,195,762	11.29
13	1,662,935,723,189	13.14	13	1,885,131,153,122	12.41

**Table A9:** FCC lattice, tube cross section $3.0\sqrt{2}\times 3.0\sqrt{2}$.

	**Type 1**	**Multiplicity |*O*_1_| = 4**		**Type 2**	**Multiplicity |*O*_2_| = 8**
***N***	***c_N_***	$\langle {\left|\omega^{N}\right|}^{2}\rangle$	***N***	***c_N_***	$\langle {\left|\omega^{N}\right|}^{2}\rangle$
1	5	1.000	1	8	1.000
2	39	2.256	2	72	2.222
3	317	3.738	3	637	3.474
4	2707	5.402	4	5683	4.881
5	23,701	7.209	5	50,802	6.330
6	211,575	9.140	6	455,104	7.820
7	1,903,598	11.06	7	4,070,009	9.286
8	17,110,652	12.87	8	36,207,759	10.67
9	152,867,156	14.52	9	319,799,348	11.95
10	1,354,729,516	16.02	10	2,803,337,706	13.14
11	11,906,603,784	17.38	11	24,402,025,435	14.26
12	103,849,402,452	18.63	12	211,104,465,801	15.32
13	899,747,181,304	19.79	13	1,816,626,021,973	16.35
	**Type 3**	**Multiplicity |*O*_3_| = 4**		**Type 4**	**Multiplicity |*O*_4_| = 4**
***N***	***c_N_***	$\langle {\left|\omega^{N}\right|}^{2}\rangle$	***N***	***c_N_***	$\langle {\left|\omega^{N}\right|}^{2}\rangle$
1	12	1.000	1	12	1.000
2	101	1.941	2	116	2.069
3	847	3.116	3	1100	3.313
4	7365	4.472	4	10,076	4.478
5	65,563	5.968	5	90,588	5.648
6	587,910	7.447	6	806,164	6.802
7	5,257,852	8.837	7	7,114,248	7.907
8	46,707,884	10.13	8	62,314,664	8.960
9	411,696,828	11.33	9	542,275,908	9.972
10	3,601,355,396	12.46	10	4,692,529,524	10.96
11	31,287,972,228	13.53	11	40,409,930,416	11.93
12	270,207,494,804	14.57	12	346,527,771,156	12.90
13	2,321,640,993,718	15.59	13	2,960,543,277,900	13.89
	**Type 5**	**Multiplicity |*O*_5_| = 4**		**Type 6**	**Multiplicity |*O*_6_| = 1**
***N***	***c_N_***	$\langle {\left|\omega^{N}\right|}^{2}\rangle$	***N***	***c_N_***	$\langle {\left|\omega^{N}\right|}^{2}\rangle$
1	12	1.000	1	12	1.000
2	132	2.182	2	132	2.182
3	1277	3.249	3	1404	3.496
4	11,839	4.380	4	13,680	4.530
5	107,062	5.466	5	125,376	5.383
6	950,202	6.476	6	1,109,776	6.157
7	8,326,206	7.429	7	9,637,976	6.915
8	72,328,430	8.352	8	82,849,936	7.690
9	624,508,830	9.265	9	708,279,448	8.499
10	5,368,075,614	10.18	10	6,035,931,488	9.350
11	45,975,770,236	11.12	11	51,329,173,080	10.25
12	392,534,289,628	12.07	12	435,731,432,064	11.19
13	3,341,824,209,214	13.06	13	3,692,543,313,752	12.19
